# Damage Identification in Composite Wind Turbine Blades Using Relative Natural Frequency Changes and Bayesian Probability

**DOI:** 10.3390/ma18235263

**Published:** 2025-11-21

**Authors:** Panida Kaewniam, Qingyang Wei, Haoan Gu, Nizar Faisal Alkayem, Maosen Cao

**Affiliations:** 1 College of Mechanics and Engineering Science, Hohai University, Nanjing 211100, China; panida.kae@egat.co.th (P.K.); mechgha@hhu.edu.cn (H.G.); cmszhy@hhu.edu.cn (M.C.); 2 Renewable Energy Planning and Feasibility Study Department, Electricity Generating Authority of Thailand, Nonthaburi 11130, Thailand; 3 College of Mechanical and Electrical Engineering, Hohai University, Changzhou 213200, China; weiqingyang@hhu.edu.cn; 4 College of Automation, Nanjing University of Posts and Telecommunications, Nanjing 210046, China

**Keywords:** wind turbine blade, composite material, modal analysis, relative natural frequency change, Bayesian probability, damage detection

## Abstract

Structural health monitoring (SHM) of composite wind turbine blades (WTBs) is crucial for improving power efficiency, reducing maintenance costs, and ensuring long-term structural reliability. Traditional frequency-based damage detection, often derived from simplified isotropic beam principles, can be challenged by the anisotropy, heterogeneity, and geometric complexity of composite WTBs. Moreover, as global indicators, natural frequencies are sensitive to environmental variations but are also limited in localizing damage. To overcome these challenges, this research introduces a combined approach of relative natural frequency change (RNFC) and Bayesian probability, referred to as the B-RNFC method. The framework includes four stages: (i) analyzing the correlation between natural frequencies and damage conditions (location and severity) in composite cantilever beams and WTBs; (ii) developing normalized RNFC curves from various damage sizes to establish a spatial damage reference dataset, which is then used for the next steps; (iii) integrating the resulting frequency-related data with Bayesian probability to identify damage locations and map them onto the structures; and (iv) evaluating the performance of the B-RNFC in multiple-damage localization. Simulation results demonstrate the effective damage localization range of the B-RNFC method. For a simple cantilever beam, this range is 20–80% of the distance from the fixed end. When applied to the composite WTB, this effective range corresponds to 40–80% of the blade length from the root. In addition, the proposed method can localize the dual damages when the damages are symmetrically located or when one damage is at the mid-span.

## 1. Introduction

Wind turbines are becoming a standard means of generating clean energy. The most sensitive parts of them are the blades, as they are the moving parts and are affected by wind gusts [1]. Blade inspection is a necessary process to prevent serious accidents. Many non-destructive methods are used to evaluate the structural conditions of the wind turbine blades by means of local damage identification techniques, such as ultrasonic testing [2,3,4], infrared thermography [5,6,7], radiography (X-ray) [2], thermography testing [8,9], acoustic emission [10,11], and so on. Another traditional nondestructive method, a global damage identification technique, is vibration-based. Vibration-based damage identification requires an excitation to impulse the structural system, after which the measured output, representing the dynamic response, is recorded and extracted to interpret the structural conditions. In other words, this structural response can be analyzed to detect the damage.

### 1.1. Background of Modal Analysis in Structural Health Monitoring

Vibration-based structural damage identification methods are traditional techniques for monitoring the health of a structure and detecting long-term global damage. The basic principle of these methods is to evaluate changes in the vibration characteristics of structures. The vibration characteristics of structures depend on their physical properties. Changes in physical properties from the intact state indicate structural damage. Therefore, the physical properties are indicators of the health conditions of the structure, including the presence, location, and severity [12,13].

The oldest vibration-based methods for identifying damage are modal-based methods [14]. As previously mentioned, changes in vibration responses are related to structural properties, including mass, damping, and stiffness. These physical changes are performed in terms of modal characteristics (natural frequencies, modal damping, and mode shapes), which is called modal analysis [15]. Moreover, modal analysis can be classified into two main subcategories: experimental modal analysis (EMA) and operational modal analysis (OMA). The EMA technique uses artificially forced excitations, such as an impact hammer and a shaker, to excite the structure, while the OMA is performed by ambient excitation as an unknown input [16].

The structural damage, such as cracks, delamination, material degradation, or stiffness reduction, results in changes to modal parameters, including natural frequencies, mode shapes, and damping ratios. It compares modal parameters between healthy and damaged states to evaluate their deviations. Many researchers developed damage-detecting methods based on modal parameters. For example, Chinka et al. [17] identified cracks in a cantilever beam using natural frequencies, mode shapes, and mode shape curvatures. Their research found that cracks reduced structural stiffness, thereby lowering natural frequencies. Mode shape curvature was the sensitive parameter for localizing crack locations. Next, Sha et al. [18] proposed a novel frequency-based method that used RNFC curves and probability in data fusion to detect single/multiple damages on the fixed-fixed beam. Their study showed that damage could be detected and localized by only natural frequency requirements. In similar research, Gillich et al. [19] developed RNFCs using the superposition principle to detect multiple cracks in a beam. Their analysis revealed that the distance between two cracks influenced damage detectability, and structural mode shapes were necessary for their analysis. According to another study by Gillich and Praisach [20], a novel damage identification method was proposed that uses time-frequency analysis and power spectrum analysis. Their study emphasized that damage occurrence and location could be observed from changes in natural frequency and signal characteristics, respectively. In the research by Gorgin [21], a damage index was presented to identify beam damage by analyzing the first mode shape. This proposed method utilized the beam’s geometrical parameters and material properties, as they were sensitive to abrupt changes in the damage index. Their results emphasized that this method could localize single/multiple damage events, including estimation of damage size and severity. Páleník et al. [22] introduced a beam’s damage detection based on modal characteristics. Their method compared the changes in natural frequency to the curvature of the mode shape and analyzed them using polynomial regression. This research could localize and quantify the damage in both 1D and 2D problems. Yang and Oyadiji [23] proposed a multiple-mode damage indicator by analyzing the modal frequency curve and wavelet transform. Their study could localize damage and estimate its extent, with greater accuracy than traditional wavelet transforms. In another advanced research study, Wei et al. [24] proposed a novel method for identifying time-varying modal parameters, known as lightweight stochastic subspace identification. Their developed technique provided more efficient computational performance and enabled better monitoring of changes in structural dynamic properties. From the effectiveness of modal analysis in SHM, it has been applied to identify damage in various civil structures such as beams [17,25,26,27,28], bridges [29,30,31], buildings [32,33,34], masonry structures [35,36,37], and tower structures [38]. Moreover, the modal parameters were integrated with various techniques, such as wavelet transforms [39,40,41], model updating [42,43], and others, to enhance damage detectability in SHM.

### 1.2. Current Status of Modal-Based Damage-Detecting Methods for Wind Turbine Blades

The application of modal-based methods has also been extended in the wind energy industry. Much previous research has employed them to identify damage on wind turbine structures (not limited to WTBs). For example, Oliveira et al. [44] analyzed the 1-year-measured acceleration data of wind turbine towers through the operational modal analysis, showing the 1D mode shapes. In another research by Oliveira et al. [45], they analyzed the recorded data from a 2.0-MW wind turbine using OMA. The results were presented in terms of natural frequencies, mode shapes, and damping ratios, which enhanced understanding of the dynamic behaviors of onshore wind turbines. In a similar study, Sellami et al. [46] analyzed the dynamic responses of the main wind turbine structure (e.g., tower, blade, and nacelle) based on modal characteristics. The results were demonstrated in terms of the modal features of each component. In another work, Adams et al. [47] used statistical pattern recognition to analyze operating loads and damage types. The obtained results, including natural frequencies and deflection shapes, were used to estimate the blade’s physical sensitivity.

Although modal-based methods have been used to investigate damage to wind turbines, research on WTBs has been limited and warrants further exploration. Some existing studies include that done by Ou et al. [48], who extracted the modal properties from a small-sized blade, such as natural frequencies, damping ratios, and mode shapes. They were used to detect damage through the vibration-based experimental method. Their results indicated that modal damage detection was a potential technique only to a certain extent. For example, the damage may be detectable in the natural frequency, but it may not be clearly visible in the mode shape of the same vibration mode. In related research, Doliński and Krawczuk [49] localized the damage on small-scale WTBs by using modal parameters. The experimental mode shapes revealed that the results for healthy and damaged blades were significantly different; however, the mode shapes were unable to localize the damage. In another advanced research, Ulriksen et al. [50] introduced the OMA and wavelet transformation to detect a trailing-edge debonding of a 34 m long blade under running conditions. The findings were presented as a mode shape and the modal assurance criteria (MAC), a basic damage predictor, to define damage. According to the experiments, the proposed method detected and localized the 1.2 m trailing-edge debonding in the eight modes. Additionally, excitation input and mechanical models are not required for analysis.

According to the literature, using a single parameter, either natural frequencies or mode shapes, is not sufficiently efficient for precisely detecting and identifying damage. For example, natural frequencies change with damage, but there is no potential for spatial resolution. Similarly, mode shapes can clearly highlight differences between healthy and damaged states when the damage is significant enough. Therefore, a combination of modal parameters or other methods can improve the damage detectability.

Natural frequency-based damage detection generally relies on evaluating frequency changes between healthy and damaged states, caused by alterations in structural stiffness or mass distribution. Because natural frequencies are global properties of a structure, these approaches are insensitive to local damage, which produces only minor shifts in frequency. Moreover, similar frequency changes may occur at different damage locations, leading to ambiguity in localization. Natural frequencies are also highly influenced by environmental and operational variations—such as temperature, humidity, boundary constraints, and aerodynamic loading—which can induce greater frequency shifts than those caused by minor damage. This limitation is particularly critical for WTBs operating under diverse environmental conditions. Although several frequency-based approaches, including frequency ratio, frequency change percentage, frequency response function, and relative natural frequency change (RNFC), have been developed to improve accuracy, their application to WTB structures remains under investigation. Therefore, this research proposes an integration method combining relative natural frequency change (RNFC) and Bayesian probability, referred to as the B-RNFC method.

### 1.3. Research Gaps and Contributions

According to the literature mentioned above, the research gaps are summarized as follows:

- Even though much existing research has analyzed behaviors of natural frequency under different damage conditions on simple geometries (e.g., beams), there are limited investigations into complicated structures like WTBs. Moreover, comparative studies of natural frequency characteristics in damaged structures across analytical techniques and numerical simulations are lacking.
- Most natural frequency-based damage detection methods have been developed and validated on simple structures, such as beams or plates. However, their applicability to complex, non-uniform geometries, like WTBs, remains limited. The transition from idealized models to realistic structures requires further investigation.
- Initially, the B-RNFC method was first applied to a fixed-fixed beam. It successfully identified the structural damage. However, using the B-RNFC method to cantilever beams and fixed-free WTBs is more challenging.
- In the beginning state of beam damage detection, calculation of the normalized RNFC curve—which serves as a spatial damage reference dataset in the B-RNFC method—does not account for damage size. For real-world structures, the effect of damage size on this curve must be investigated.
- Based on existing research, the B-RNFC method can detect damage at specific locations, but does not quantify and map the detectable locations along the structures. Moreover, this spatial sensitivity of the B-RNFC method has not been discovered for the WTBs.

To address the aforementioned research gaps, this article introduces a damage identification method for WTBs using the B-RNFC method. The main contributions of this study are condensed as follows:

- The research provides a comparative analysis of the relationship between natural frequencies and damage conditions (damage severities and locations) of cantilever beams and WTBs. Moreover, the obtained simulation results are verified through analytical analysis.
- This research extends the B-RNFC method to complex WTBs. It demonstrates applicability to realistic structures, highlighting the pros and cons of this proposed method.
- The normalized RNFC curves from different damage sizes are introduced to address the limitation of prior research and to provide a more robust understanding of reference parameters for damage detection in real structures.
- This study systematically varies damage locations along the cantilever beams and blades and discovers the detectable damage locations. It provides the B-RNFC’s strengths and blind spots in practical implementation.

## 2. Formulation of the B-RNFC Method

In this research, a damage detection method using natural frequency, referred to as the B-RNFC method, is proposed. The B-RNFC is a fusion of relative natural frequency changes (RNFC) and Bayesian probability. Moreover, the probabilistic damage indicator (PDI) is introduced in the B-RNFC analysis to localize the structural defects.

The core concept of the B-RNFC method is provided for foundational understanding. This proposed method requires two data components: normalized RNFC curves and normalized RNFC values. The analysis begins by generating a spatial damage reference dataset, known as normalized RNFC curves, through the simulation of artificial damage and the extraction of natural frequencies at each node/location along the structure. Changes in relative natural frequencies across multiple modes serve as reference data linking frequency shifts to damage positions. The second component, the normalized RNFC values, is derived from the measured frequencies of the damaged structure. The difference between the normalized RNFC curves and the normalized RNFC values is quantified using the Damage Position Function (DPF), which identifies the most probable damage location. To enhance reliability, the Bayesian probabilistic framework is incorporated to interpret the DPF results in terms of the probability of damage occurrence. This Bayesian principle can quantify uncertainty and enhance the robustness of decision-making in the face of modeling inaccuracy by utilizing the Probabilistic Damage Indicator (PDI). The overall B-RNFC process framework is illustrated as follows:

### 2.1. Normalized RNFC Curves and Normalized RNFC Values

Based on preliminary assumptions, a cantilever beam is analyzed using the B-RNFC damage investigation. In this study, the structural damage is modeled by reducing the material stiffness. The B-RNFC method is a reference-based damage detection method that needs a reference dataset and actual damage data. Therefore, the reference data is expressed as the normalized RNFC curve at a constant stiffness reduction, while the actual damage data is calculated based on the normalized RNFC values.

**Figure 1:**
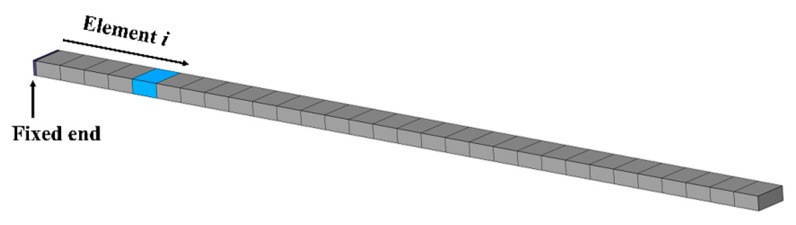
Cantilever beam with damage located at element i.

First, the normalized RNFC curve is computed at a specific stiffness reduction. From Figure 1, the damage created in the element i is denoted as a normalized damage location i ($\zeta_{i}$). The $\zeta_{i}$ is calculated from ${\zeta_{i}=l}_{i}/L$, where $l_{i}$ and L are the local damage location i and the total structural length, respectively. The natural frequency of the intact structure at j^th^ mode is $F_{j}$. The j^th^ natural frequency of the damaged structure at location i is defined as $F_{i,j}^{d}$. The RNFC caused by structural damage is quantified in Equation (1), denoted as ${\Delta F}_{i,j}$.

(1) $${\Delta F}_{i,j}=\frac{F_{j}-F_{i,j}^{d}}{F_{j}}$$

At a constant damage severity, the $j^{th}$ natural frequency change of location i (${\Delta F}_{i,j}$) is normalized to the range 0–1. The normalized RNFC curve ($\Delta {\bar{F}}_{i,j}$) is calculated to compare across all damage positions, as mentioned in Equation (2).

(2) $$\Delta {\bar{F}}_{i,j}=\frac{\Delta F_{i,j}-{min}_{i}(\Delta F_{i,j})}{{max}_{i}\left(\Delta F_{i,j}\right)-{min}_{i}(\Delta F_{i,j})}$$

According to the $\Delta {\bar{F}}_{i,j}$ only related to damage locations, the normalized RNFC curve can also be expressed as

(3) $$g_{j}(\zeta_{i})=\Delta {\bar{F}}_{i,j}$$

Next, another calculation is the normalized RNFC values from the actual defect. The j^th^ natural frequencies of intact and damaged structures are $f_{j}$ and $f_{j}^{d}$, respectively. The measured RNFC value (${\Delta f}_{j}$) can be computed as Equation (4).

(4) $${\Delta f}_{j}=\frac{f_{j}-f_{j}^{d}}{f_{j}}$$

Likewise, the ${\Delta f}_{j}$ is normalized across the mode j and is denoted as:

(5) $$\Delta {\bar{f}}_{j}=\;\frac{\Delta f_{j}-min(\Delta f_{j})}{max\left(\Delta f_{j}\right)\;-min(\Delta f_{j})}$$

### 2.2. Damage-Localizing Algorithms

After obtaining the normalized RNFC curve and normalized RNFC values, the frequency-related comparison between the baseline data and actual damage is expressed as the damage position function (DPF). The DPF at the damage location i of the j^th^ mode is obtained as:

(6) $${DPF}_{i,j}=1-\left|g_{j}(\zeta_{i})\;-\;\Delta {\bar{f}}_{j}\right|$$

Statistical methods, such as Bayesian theory, are applied to combine multiple DPFs. The Bayesian theory is a potential tool for data fusion in the area of damage detection [51,52,53]. The general form of Bayesian theory is explained in Equation (7). The common idea of this approach is to update the probability of a hypothesis based on new evidence. The $A_{i}$ is a hypothesis of the possible damage location i along the structure, which is n positions as $A_{1},\;A_{2},.\;..,\;A_{n}$. The $P(A_{i})$ represents the prior probability from the initial hypothesis. The measured data of each $j^{th}$ mode is called the $D_{j}$, and there are m modes (j=1, 2,…,m). The measured data is denoted as $D_{1},\;D_{2},.\;..,\;D_{m}$. The term of $P(D_{1},D_{2},\ldots ,D_{m}|A_{i})$ is a conditional probability of a hypothesis $A_{i}$, which is observed in the data $D_{j}$ if the damage exists at $A_{i}$. In another meaning, if the damage is at the location $A_{i}$, how likely would we see this data $D_{j}$?

(7) $$P\left(A_{i}\right|D_{1},D_{2},\ldots ,D_{m})=\frac{P\left(D_{1},D_{2},\ldots ,D_{m}|A_{i}\right)\cdot P(A_{i})}{\sum_{u=1}^{n}\left(P(D_{1},D_{2},\ldots ,D_{m}|A_{u}\right)\cdot P(A_{u}))}$$

Because the data observations $D_{1},D_{2},\ldots ,D_{m}$ are independent under the condition of $A_{i}$, their combination uses a product operation described as Equation (8).

(8) $$P(D_{1},D_{2},\ldots ,D_{m}|A_{i})=\prod_{j=1}^{m}P(D_{j}|A_{i})$$

Then, the Bayesian data fusion is derived by substituting Equation (8) into Equation (7)

(9) $$P\left(A_{i}\right|D_{1},D_{2},\ldots ,D_{m})=\frac{\prod_{j=1}^{m}P(D_{j}|A_{i})\cdot P(A_{i})}{\sum_{u=1}^{n}\;(\prod_{j=1}^{m}P(D_{j}|A_{u})\cdot P(A_{u}))}$$

Assume that each damaged location i has an equal chance of causing real structural damage. Then, the prior probability is equivalent to $P\left(A_{i}\right)=1/n$. The conditional probability of $A_{i}$ is implied to ${DPF}_{i,j}$ as shown in Equation (10).

(10) $$P\left(D_{j}|A_{i}\right)={DPF}_{i,j}$$

Substitute Equation (10) into Equation (9), and is expressed as Equation (11).

(11) $$P\left(A_{i}\right|D_{1},D_{2},\ldots ,D_{m})=\frac{\prod_{j=1}^{m}{DPF}_{i,j}\cdot P(A_{i})}{\sum_{u=1}^{n}\;(\prod_{j=1}^{m}{DPF}_{u,j}\cdot P(A_{u}))}$$

From Equation (11), the common terms are neglected as

(12) $$P\left(A_{i}\right|D_{1},D_{2},\ldots ,D_{m})=\frac{\prod_{j=1}^{m}{DPF}_{i,j}}{\sum_{u=1}^{n}\;(\prod_{j=1}^{m}{DPF}_{u,j})}$$

So far, the obtained Bayesian probability can predict the possible location of damage. If the natural frequencies used in the above calculation are disturbed by noise, the Bayesian probability may not express accurate results. Therefore, a post-processing step of the Bayesian probability, which is based on the principle of structural symmetry and geometric mean operation, is applied to reduce imperfection or noise effect [18]. The Bayesian probability of the candidate location i, in the form of $P\left(A_{i}\right|D_{1},D_{2},\ldots ,D_{m})$, is simplified as $P_{i}$. While the probability of its symmetric location i is $P_{n+1-i}$. The improved probability ($Q_{i}$) is denoted as:

(13) $$Q_{i}=\sqrt{P_{i}P_{n+1-i}}$$

Moreover, the symmetry-improved probability emphasizes the damage location and eliminates any background noise by Z-score normalization, as in Equation (14).

(14) $$Z(Q_{i})=\frac{Q_{i}-mean(Q)}{SD(Q)}$$

where the SD(Q) is the standard deviation of $Q_{i}$.

Lastly, the probabilistic damage indicator (PDI) in localizing the damage is calculated as given in Equation (15). The peak of PDI represents the location of damage.

(15) $${PDI}_{i}=\left\{\begin{array}{c} Z\left(Q_{i}\right),\;\;if\;Z\left(Q_{i}\right)\geq 0\;\\ 0,\;\;\;\;\;\;\;\;\;\;\;\;\;\;\;\;if\;Z\left(Q_{i}\right)<0 \end{array}\right.$$

### 2.3. Research Procedure

**Figure 2:**
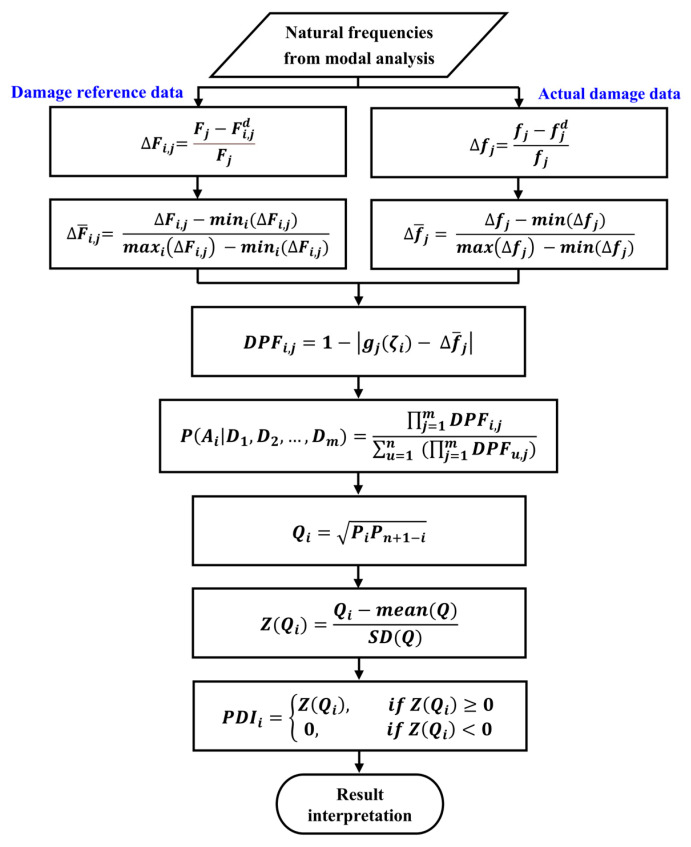
The B-RNFC method to detect structural damage.

Damage detection using the B-RNFC method requires natural frequency data to first calculate the normalized RNFC curves and normalized RNFC values. Then, the differences between the normalized RNFC curves and RNFC values are evaluated as the damage position function (DPF). Finally, these DPFs are fused by Bayesian probability to calculate the probabilistic damage indicator (PDI). The damage investigation through the B-RNFC method is explained in Figure 2.

In this research, the frequencies of the first five bending modes are used in this analysis. According to existing studies, the B-RNFC method was applied to the fixed-fixed beam [18]. Therefore, the proposed algorithm is first verified using their results, and the latter step involves analyzing the cantilever beam and fixed-free blade. Theoretically, the normalized RNFC curves for beam structures at constant damage severity are calculated from the frequencies of all damage locations, thereby ignoring the effect of damage size. In practical applications of WTBs, the damage sizes used to establish the RNFC curve must be considered. Then, the different damage sizes are used to construct normalized RNFC curves and to analyze their effects on damage detection results.

The research on the B-RNFC method is divided into four parts. First, the relationship between natural frequencies and damage conditions (damage locations and severities) for cantilever beams and WTBs is studied. Next, the normalized RNFC curves from various damage sizes are analyzed to understand the effect on damage detection. Then, the performance of the B-RNFC technique for damage detection in both beams and blades, including the detectable damage locations, is investigated. Finally, an analysis of multiple-damage scenarios is conducted based on the detectable range of single damage.

As previously explained, this study focuses on evaluating a key capability of the B-RNFC method: damage localization. The research framework is structured into two main localization tasks: (i) establishing the detectable range for a single damage on both a cantilever beam and a fixed-free WTB, and (ii) evaluating the method’s ability to identify multiple damages at crucial points selected based on structural symmetry. Therefore, this potential assessment is a crucial investigation for use in complex structural scenarios.

## 3. Numerical Simulation and Case Studies

### 3.1. Numerical Simulation

**Figure 3:**
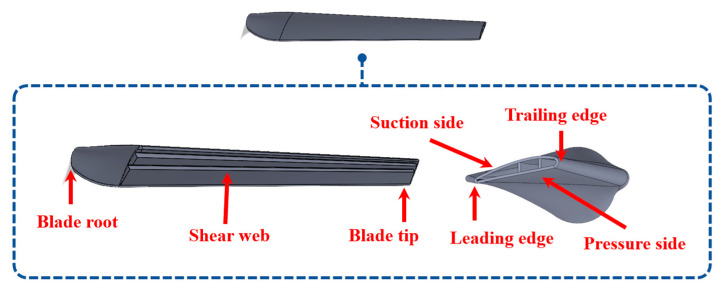
Structure of the wind turbine blade used in this study.

The WTBs are complicated structures designed differently to maximize energy production. Their manufacture utilizes composite materials, including fiberglass and carbon fiber. In existing research, WTBs were modeled using shell elements [54,55] or simplified as beams/one-dimensional (1D) shapes [56]. To approach the real structure, this study proposes a three-dimensional (3D) WTB with solid elements. It is designed based on the NACA4411-63 profile with a 1.2 m length and 0.0026 m thickness. The maximum and minimum chord lengths are 0.16 and 0.087 m, respectively. The considered area of damage creation is on the airfoil section, which is between 0.2 and 1.2 m from the blade root (or ζ is between 0 and 1). The modeled WTB used in this research is shown in Figure 3.

In the numerical simulation, the blade is fixed at its root and meshed with hexahedral elements. The composite material used in this study is Epoxy Carbon UD Prepreg, which is from the ANSYS Material Library 2022 R1 [57]. Due to the anisotropic properties, it is converted to match the model direction, as described in Table 1, and its layers are ignored. The modal analysis is conducted by the Block Lanczos method through the ANSYS APDL 2022 R1 program. This research creates structural damage by stiffness reduction, and its details are explained in the next section.

### 3.2. Damage Setup

As mentioned in the research procedure section, the relationship among natural frequencies, damage locations, and damage severities (or stiffness reductions) for cantilever beams and WTBs is comprehensively analyzed. Damage is modeled by reducing the stiffness of elements in the target area. The reduction is applied in 2% increments, ranging from 0% to 50%. Damage locations are defined at every node along the blade’s span. Each location consists of a set of elements connected to that node. After obtaining the simulation results, they are validated by comparing them with the analytical model results.

Next, normalized RNFC curves are analyzed for different damage sizes (1.5 × 1.5 cm, 3.5 × 1.5 cm, and 5.0 × 1.5 cm). In the absence of prior research on damage size effects for RNFC curves, these dimensions are chosen relative to the mesh element size to represent the most minor detectable damage. In all cases, damage was simulated as a 30% reduction in stiffness, a moderate level between moisture-induced degradation and extreme damage [39]. The damage locations are varied between 0.2 and 1.2 m from the fixed end (ζ is ranked from 0 to 1). For the calculation of normalized RNFC values, the actual damage location is expressed as a normalized damage location (ζ). In this research, the RNFC values are set to 0.5 and 0.7, as these represent common damage locations on a blade [39].

Then, the performance of the B-RNFC method in the WTB damage detection is analyzed by varying actual damage locations along the blade (from ζ = 0.1–0.9, every 0.1 increment). The B-RNFC detectability of WTBs is also compared with that of cantilever beams.

Following the single-damage analysis, the evaluation of the B-RNFC method is extended to multiple damage localization on the cantilever beam. The multiple damage cases are confined to the single-damage detectable range, and with each damage is simulated by a 30% stiffness reduction. Three multiple-damage configurations are analyzed: (i) damages at ζ = 0.3 and 0.5, (ii) damages at ζ = 0.2 and 0.8, and (iii) damages at ζ = 0.2 and 0.6. These configurations are designed according to the structural symmetry principle.

## 4. Results and Discussions

Analysis of the B-RNFC method and related issues have been conducted for four stages: (i) analyzing the correlation between natural frequencies and damage conditions (locations and severities) in composite cantilever beams and WTBs; (ii) developing normalized RNFC curves from various damage sizes to serve as spatial damage reference data, and employing the suitable RNFC curve for damage localization in the next steps; (iii) integrating the proficient curve with Bayesian probability to identify damage locations and map on the structures; and (iv) evaluating the performance of the B-RNFC in multiple-damage localization.

### 4.1. Relationship Between Natural Frequencies and Damage Conditions

Research on the relationship between natural frequencies and damage parameters for a cantilever beam and a fixed-free blade is conducted. The damage is characterized by stiffness reduction (SR) and normalized damage location (ζ).

**Figure 4:**
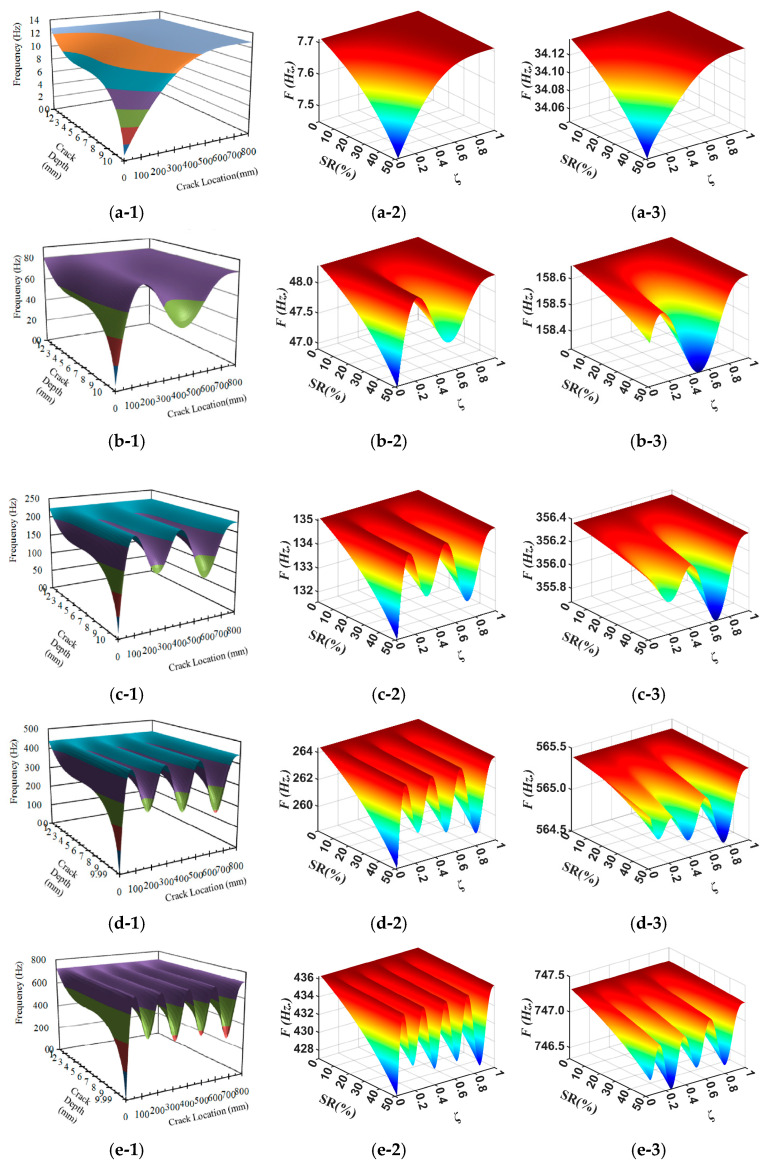
The relationship among natural frequencies, stiffness reduction, and damage locations. The first, second, and third columns represent the analytical technique for the beam [17], simulation for the beam, and simulation for the blade, respectively. (**a-1**–**a-3**) mode 1, (**b-1**–**b-3**) mode 2, (**c-1**–**c-3**) mode 3, (**d-1**–**d-3**) mode 4, and (**e-1**–**e-3**) mode 5.

First, the natural frequencies of the beams obtained from the simulation are compared with the analytical results from [17], as shown in the first and second columns of Figure 4. Although the beam dimensions and material properties differ between the numerical model and the analytical technique, leading to different absolute frequency values, the patterns of natural frequency changes with damage conditions are consistent. This agreement serves as a preliminary validation of the simulation methodology.

Subsequently, the natural frequencies of the beam and blade are extracted from simulations, with results presented in the second and third columns of Figure 4. The frequency patterns of the blade differ from those of the beam, particularly near the root (ζ=0−0.2). Owing to limitations in modeling damage at the blade root, the analyzed damage region was confined to ζ=0.2−1.0. Despite this limitation, the extracted blade frequencies still effectively capture the relationship between natural frequencies and the damage parameters.

As shown in Figure 4, the natural frequencies of the cantilever beam and WTB, especially in the first mode, decrease as damage severity increases. Conversely, the relationship between natural frequencies and damage locations is highly mode-specific, with no single consistent trend.

### 4.2. Study of Normalized RNFC Curves Under Different Damage Sizes

Normalized RNFC curves are a spatial damage reference dataset generated by simulating damage at each node along the structure and extracting the natural frequencies for all bending modes. The primary application of the B-RNFC method on a beam did not account for damage size in the normalized RNFC curves. However, for a real-world structure such as a WTB, selecting the appropriate damage size is critical and requires thorough investigation. Therefore, the normalized RNFC curves are analyzed for three different damage sizes (2.0 × 1.5 cm, 3.5 × 1.5 cm, and 5.0 × 1.5 cm), each modeled with a 30% reduction in stiffness.

**Figure 5:**
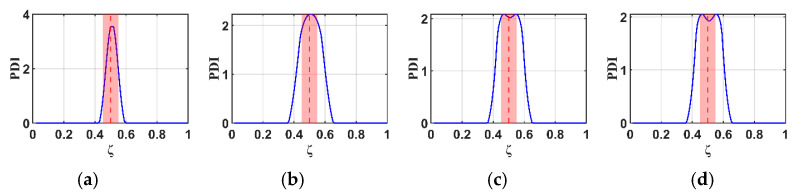
Comparison of B-RNFC results for an actual damage location at 0.5 (dashed line), showing different normalized RNFC curves from: (**a**) a cantilever beam, as simplified shape, and from a blade with simulated damage sizes of (**b**) 2.0 × 1.5 cm, (**c**) 3.5 × 1.5 cm, and (**d**) 5.0 × 1.5 cm.

**Figure 6:**
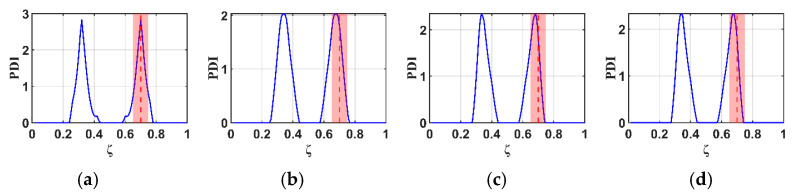
Comparison of B-RNFC results for an actual damage location at 0.7 (dashed line), showing different normalized RNFC curves from: (**a**) a cantilever beam, as simplified shape, and from a blade with simulated damage sizes of (**b**) 2.0 × 1.5 cm, (**c**) 3.5 × 1.5 cm, and (**d**) 5.0 × 1.5 cm.

To study the effects of the RNFC curves in this proposed method, normalized RNFC values are calculated for normalized damage positions (ζ) at 0.5 and 0.7. The B-RNFC results for damage identification in the WTBs, based on the parameters mentioned above, are shown in Figure 5 and Figure 6.

According to the theory of the B-RNFC method, the damage-detecting outcomes exhibit symmetric peaks, corresponding to the actual damage location and its false counterpart. This is a common phenomenon from the probability improvement in Equation (13). Even though the results show symmetric peaks, they narrow down to two possible damage locations. The B-RNFC results in Figure 5, corresponding to a normalized damage location (ζ) of 0.5, show a single PDI peak at the mid-span for both structures. This is because the actual damage is located at its own mirror point of structural symmetry. Conversely, for damage at ζ=0.7, the results show the symmetric PDI peaks, as shown in Figure 6. Lastly, the simulation results from both beam and blade structures are validated against a previous study [18] that analyzed the B-RNFC method on a fixed-fixed beam. It is shown that the PDI peak locations accurately identify damage at both mid-span and non-mid-span positions. The trends observed in this study align with those reported in prior research.

For the cantilever beams, the B-RNFC method can accurately localize the damage at 0.5 and 0.7, as shown in Figure 5a and Figure 6a. The PDI peak is at the same location as the actual damage (dashed line). This implies that the B-RNFC method is efficient at detecting damage in beam structures.

For the fixed-free WTBs, the damage sizes in forming normalized RNFC curves slightly affect the PDI peaks, where the actual damage is at 0.5, as shown in Figure 5b–d. Likewise, the PDI peaks of all normalized RNFC curves in Figure 6b–d are near 0.7. To set the detectable range, the acceptance distance from the actual damage location is 0.05 m on both the left and right sides (red area between dashed line). Therefore, the normalized RNFC curves from the three damaged samples express almost identical results. It implies that the size of damage in establishing RNFC curves does not significantly affect the damage detection results. Finally, the normalized RNFC curve from the 2.0×1.5 cm damage is selected in the latter research.

### 4.3. Effectiveness of the B-RNFC Method in Damage Detection for Wind Turbine Blades

As in the previous section, the normalized RNFC curves are first analyzed and used to establish a reference dataset. Subsequently, the B-RNFC method is applied for damage detection in beam and blade structures.

**Figure 7:**
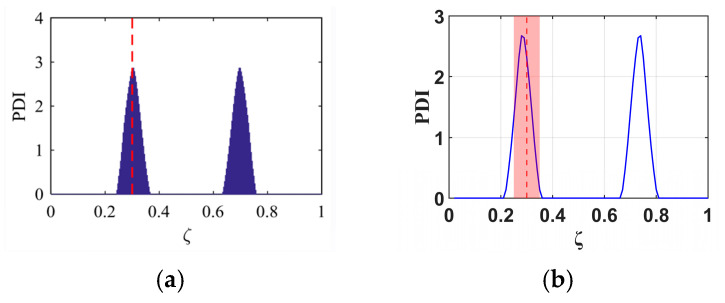
A comparison of B-RNFC results for an actual damage location at 0.3 (dashed line): (**a**) a fixed-fixed beam from the prior study [18], and (**b**) a fixed-free beam from the current study.

**Figure 8:**
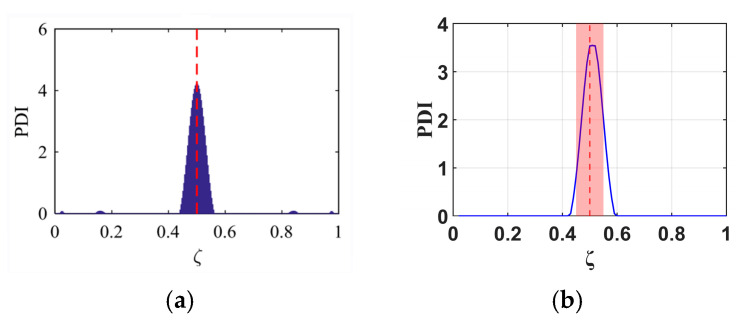
A comparison of B-RNFC results for an actual damage location at 0.5 (dashed line): (**a**) a fixed-fixed beam from the prior study [18], and (**b**) a fixed-free beam from the current study.

To validate the proposed B-RNFC method, its results are compared with those from a prior study [18], which successfully detected damage in a fixed-fixed beam at normalized positions of 0.3 and 0.5. In this research, the method is applied to a cantilever beam (fixed-free boundary condition), representing a different structural constraint. As the comparison results are shown in Figure 7 and Figure 8. The B-RNFC approach successfully identified damage at the exact locations (0.3 and 0.5) in both the previous and the current studies. The consistent performance across these differing boundary conditions demonstrates the effectiveness of the B-RNFC method for beam damage detection.

**Figure 9:**
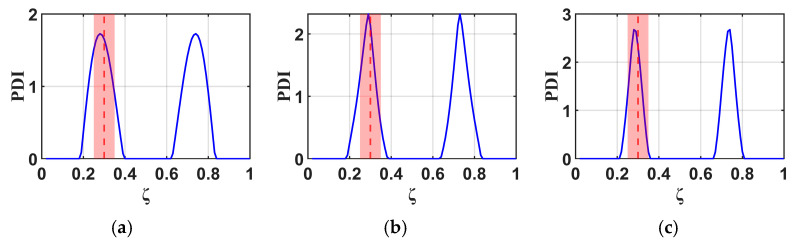
B-RNFC results for a cantilever beam with damage at location ζ = 0.3 (dashed line), analyzed using (**a**) 3 modes, (**b**) 4 modes, and (**c**) 5 modes.

**Figure 10:**
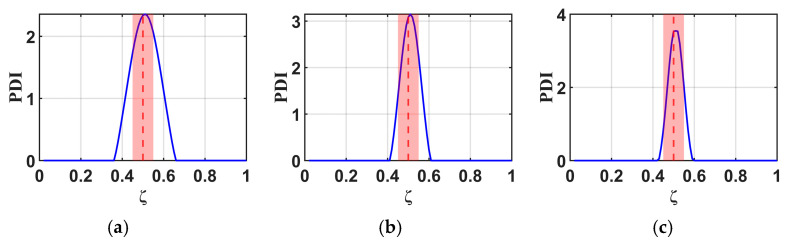
B-RNFC results for a cantilever beam with damage at location ζ = 0.5 (dashed line), analyzed using (**a**) 3 modes, (**b**) 4 modes, and (**c**) 5 modes.

**Figure 11:**
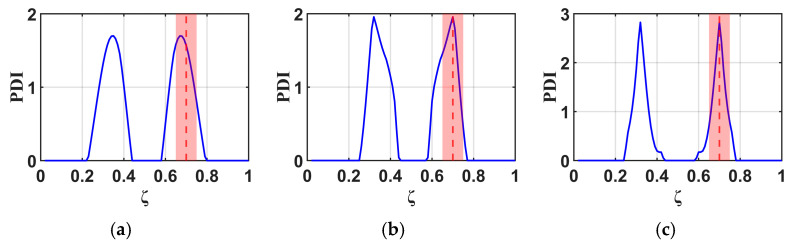
B-RNFC results for a cantilever beam with damage at location ζ = 0.7 (dashed line), analyzed using (**a**) 3 modes, (**b**) 4 modes, and (**c**) 5 modes.

According to existing research [18], the number of vibrational modes used in analysis directly influences damage detection accuracy, with more modes leading to higher accuracy. Therefore, this study identifies damage using 3, 4, and 5 vibration modes at three damage locations (ζ = 0.3, 0.5, 0.7), and the results are presented in Figure 9, Figure 10 and Figure 11. The results demonstrate that analyses incorporating a higher number of modes produce a narrower damage peak, compared to those using fewer modes. Furthermore, the peak analyzed from more mode data points precisely to the actual damage location. Therefore, the subsequent analysis in this study will utilize data from five vibration modes.

**Figure 12:**
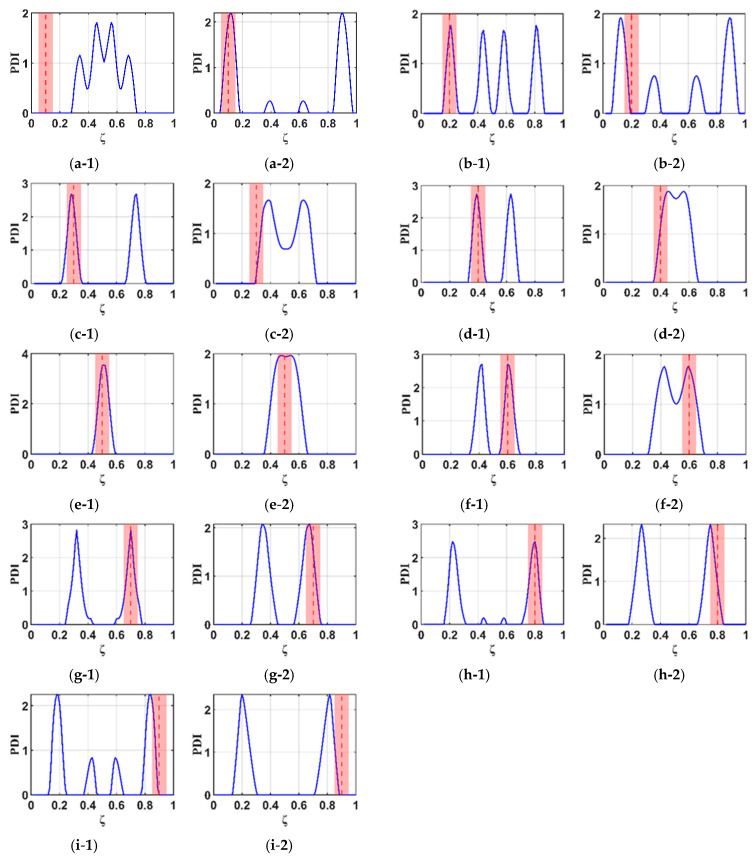
B-RNFC results corresponding to different damage locations (at dashed line). The first column presents results for beams, and the second column presents results for WTBs. The actual damage locations are (**a-1**,**a-2**) 0.1, (**b-1**,**b-2**) 0.2, (**c-1**,**c-2**) 0.3, (**d-1**,**d-2**) 0.4, (**e-1**,**e-2**) 0.5, (**f-1**,**f-2**) 0.6, (**g-1**,**g-2**) 0.7, (**h-1**,**h-2**) 0.8, and (**i-1**,**i-2**) 0.9.

In this section, the investigating performance of the B-RNFC method in different damage locations along the beams and blades is evaluated. Figure 12 presents the B-RNFC results of both structures, whose damage locations are varied every 0.1 m. For cantilever beams, the B-RNFC method can localize damage between 0.2 and 0.8 times the beam length from the fixed end. Results of all damage locations show symmetric peaks except at 0.5, as shown in Figure 12(a-1–i-1).

From Figure 12(a-2–i-2), the WTB’s damages located between 0.4 and 0.8 can be detected via the B-RNFC method (consider only the PDI peak within the acceptable clearance). The results emphasize that the B-RNFC method can potentially localize damage around the structural middle. Although the B-RNFC method can efficiently identify damage locations in cantilever beams, its application to wind blades remains limited due to geometric complexities.

### 4.4. Effectiveness of the B-RNFC Method for Multiple Damage Localization

**Figure 13:**
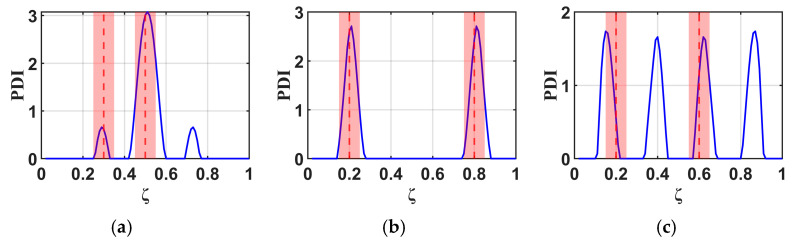
B-RNFC results for different multiple-damage scenarios: (**a**) damages at ζ = 0.3 and 0.5, (**b**) damages at ζ = 0.2 and 0.8, and (**c**) damages at ζ = 0.2 and 0.6.

Building upon the investigation of the single-damage detectable range in a cantilever beam presented in the previous section, the B-RNFC method demonstrates reliable localization capability within the region ζ = 0.2–0.8 along the beam length from the fixed end. Based on this verified range, the present section examines the effectiveness of the B-RNFC method in identifying multiple damage sites. Three multiple-damage configurations are analyzed: (i) damages at ζ = 0.3 and 0.5, (ii) damages at ζ = 0.2 and 0.8, and (iii) damages at ζ = 0.2 and 0.6. These configurations are designed according to the principle of structural symmetry. The corresponding results are illustrated in Figure 13.

Figure 13a illustrates Case 1, where multiple damages are introduced at ζ = 0.3 and 0.5, with the latter located on the mid-span of the cantilever beam. The B-RNFC method successfully identifies both damage sites. In this case, three distinct peaks are observed in the response graph: two minor peaks associated with the damage at ζ = 0.3, and one dominant peak corresponding to the damage at ζ = 0.5

Figure 13b presents Case 2, where multiple damages are located at ζ = 0.2 and 0.8, forming a symmetric configuration along the beam length. Owing to this symmetry, only two peaks appear in the response graph. Nevertheless, the B-RNFC method successfully identifies both damage locations with clear distinguishability.

Figure 13c illustrates Case 3, where multiple damages are located at ζ = 0.2 and 0.6, representing a non-symmetric damage configuration along the cantilever beam. The results indicate that the damage at ζ = 0.6 is successfully identified, with its corresponding peak appearing within the acceptable range (red region). In contrast, the peak associated with the damage at ζ = 0.2 lies outside this acceptable range

Future work will focus on determining the severity of damage. To quantify the damage-severity level after placing the damage location, several existing studies have explored the relationship between natural-frequency reduction and damage severity (in terms of stiffness degradation [58,59], damage depth [60], or equivalent damage size [61]). Generally, a greater decrease in natural frequencies is associated with higher damage severity.

## 5. Conclusions

Most natural frequency-based damage detection methods have been developed and validated on simple structures, such as beams or plates. However, their applicability to complex, non-uniform geometries, like composite WTBs, remains limited, as these methods do not fully account for the unique material behavior. To overcome these limitations, this study proposes an integrated approach of RNFC and Bayesian probability, termed the B-RNFC method, for effective damage detection in composite WTBs. Key findings of this research are summarized as follows:

- The relationship between natural frequencies and damage characteristics in composite cantilever beams and WTBs exhibits consistent trends, deviating only at the root area (ζ=0−0.2) due to limitations in the modeling of damage.
- Fundamentally, the B-RNFC method was developed based on the principle of relative frequency change applied to spatial data and the concept of structural symmetry (e.g., geometry and boundary condition). This foundation directly influences the results, as each single damage location produces symmetric peaks corresponding to the actual damage location and its false counterpart. If a single damage is located at the mid-span (mirror point), the two peaks converge and appear as a single peak.
- The proposed method demonstrates an effective damage detection range of ζ=0.2−0.8 for the cantilever beam and ζ=0.4−0.8 for the wind turbine blade. Due to the symmetry theory formulated in the B-RNFC framework, the damage detectable range of the cantilever beam (symmetric shape) is wider than that of WTB (complex geometry).
- The B-RNFC method effectively identifies multiple damages (2 damages) on the cantilever beam, particularly when the damages are symmetrically located or when one damage is at the mid-span. However, for non-symmetrical damage configurations, the method exhibited some deviation in accuracy. In this case, one damage is correctly identified, while the second damage appears outside the acceptable detection range.
- For the WTBs, the normalized RNFC curves from various damage sizes provide a slight difference in damage detection outcomes.

This study introduces a novel B-RNFC method for damage localization in cantilever beams and WTBs using only natural frequency data. The results emphasize that the technique successfully identifies a detectable range for single damage in both structural types. Furthermore, it can identify multiple damages at specific symmetric locations. These findings confirm that the proposed method is an effective tool for primary localizing damage. An advantage of the B-RNFC method is that it requires fewer sensors compared to other modal-based approaches, which is more practical for large-scale structures.

According to the new application in complex structures, such as blades, some limitations still need to be acknowledged. The proposed method uses a spatial damage reference dataset (called “a normalized RNFC curve”) derived from the natural frequencies at each node along the structure. The accuracy of damage detection improves with a high nodal density of this dataset. The method’s results are characterized by symmetric peaks, representing both the true and a mirror damage location. Therefore, the B-RNFC approach serves as a first-stage screening tool to narrow down potential damage zones. Subsequently, a complementary non-destructive testing (NDT) technique is required to pinpoint the actual damage location within these identified areas.

This research presents a preliminary study that applies the B-RNFC method to the complex geometry of WTBs. It establishes fundamental principles by addressing key challenges, including asymmetric boundary conditions, structural asymmetry, and the influence of damage size on RNFC curves. To guide future research, investigating the effects of damage severity and multiple damage sites on WTBs is essential for comprehensively enhancing the B-RNFC method for blade structures.

## Figures and Tables

**Table 1:** Material properties of Epoxy Carbon UD Prepreg [57].

Property	Value	Unit
Density	1490	kg/m^3^
Young’s Modulus (X-direction)	8.6 × 109	Pa
Young’s Modulus (Y-direction)	12.1 × 1010	Pa
Young’s Modulus (Z-direction)	8.6 × 109	Pa
Shear Modulus XY	4.7 × 109	Pa
Shear Modulus YZ	4.7 × 109	Pa
Shear Modulus XZ	3.1 × 109	Pa
Poisson’s Ratio XY	0.01919	
Poisson’s Ratio YZ	0.27	
Poisson’s Ratio XZ	0.4	

## Data Availability

The original contributions presented in this study are included in the article. Further inquiries can be directed to the corresponding author.

## Abbreviations

The following abbreviations are used in this manuscript:

B-RNFC	Bayesian-relative natural frequency change
DPF	Damage position function
EMA	Experimental modal analysis
MAC	Modal assurance criteria
NDT	Non-destructive testing
OMA	Operational modal analysis
PDI	Probabilistic damage indicator
Q	Improved probability
RNFC	Relative natural frequency change
SD	Standard deviation
SHM	Structural health monitoring
SR	Stiffness reduction
WTB	Wind turbine blade
Z	Z-score
